# Cutaneous T-Cell Lymphoma and Microbiota: Etiopathogenesis and Potential New Therapeutic Targets

**DOI:** 10.1155/2024/9919225

**Published:** 2024-02-22

**Authors:** Daniel Rodríguez Baeza, Lía Bejarano Antonio, Marta González de Arriba, José Antonio Picó-Monllor, Javier Cañueto, Vicente Navarro-Lopez

**Affiliations:** ^1^Dermatology Service, Rio Hortega University Hospital, Calle Dulzaina, 2, Valladolid 47012, Spain; ^2^MiBioPath Research Group, Medicine Faculty, Catholic University of Murcia (UCAM), Av. de los Jerónimos, 135, Murcia 30107, Spain; ^3^Dermatology Service, Salamanca University Hospital, Paseo de la Transición Española, Salamanca 37007, Spain; ^4^Faculty of Pharmacy, Department of Pharmacology, Pediatrics and Organic Chemistry, Miguel Hernández University of Elche, Ctra. Alicante-Valencia N 332, 03550 Sant Joan Alacant, Alicante, Spain; ^5^IBSAL, Institute of Biomedical Research of Salamanca, P.º de San Vicente, 182, Salamanca 37007, Spain; ^6^Clinical Microbiology and Infectious Disease Unit, Vinalopó University Hospital, c/Tonico Sansano Mora, 14, Elche 03293, Spain

## Abstract

**Objective:**

To review the scientific literature related to human microbiota and cutaneous T-cell lymphoma. *Methodology*. An exploratory and systematic review of the articles retrieved from the bibliographic databases MEDLINE (PubMed), Embase, The Cochrane Library, and Scopus, published in the last 10 years with the following descriptors: “lymphoma, T-cell, cutaneous,” “microbiota,” “Mycosis Fungoides,” “Sézary Syndrome,” “lymphoma, primary cutaneous anaplastic large cell,” “Lymphomatoid Papulosis” and “Microbiota,” “microbiota,” “Microbial Community,” and “Microbial Communities.”

**Results:**

Of the 87 references retrieved, after applying the inclusion and exclusion criteria, 21 articles were selected. Most studies linking cutaneous T-cell lymphoma and the microbiota focus on the cutaneous microbiome, with *Staphylococcus aureus* being the main related agent. Skin colonization by this bacterium could be involved in the hyperactivation of the STAT3 inflammatory pathway and in the overproduction of IL-17, both of which are widely related to the development of more aggressive and advanced forms of cutaneous T-cell lymphoma. We also found evidence of a possible relationship between intestinal dysbiosis and the development of cutaneous T-cell lymphoma, observing a decrease in taxonomic variability and an increase in certain genera such as *Prevotella* in the intestinal microbiome of patients with cutaneous T-cell lymphoma. The possible etiopathogenic mechanism underlying this relationship could be explained by an increase in systemic cytokine release, promoting the hyperactivation of STAT3 at the skin level.

**Conclusion:**

There appears to be a relationship between cutaneous T-cell lymphoma and the cutaneous and intestinal microbiome, as well as a possible pathophysiological pathway involved. The possible modulation of the cutaneous and intestinal microbiome or the action on the signaling inflammatory pathway, using pharmacological tools such as JAK inhibitors or IL-17 inhibitors in the latter case, could open the possibility for future therapeutic studies for cutaneous T-cell lymphoma.

## 1. Introduction

Cutaneous T-cell lymphoma (CTCL) is a heterogeneous group of non-Hodgkin lymphomas that originate from skin-homing T-cells and manifest as various skin lesions. CTCL is a rare disease with an incidence of 0.5–1 per 100,000 people per year. The most common subtypes of CTCL are mycosis fungoides (MF) and Sézary syndrome (SS), which account for about 60% and 5% of cases, respectively. Other less common subtypes include primary cutaneous anaplastic large cell lymphoma (pcALCL) and lymphomatoid papulosis (LyP), which belong to the spectrum of CD30-positive lymphoproliferative disorders [1].

The etiopathogenesis of CTCL remains incompletely understood, but it is likely influenced by a combination of factors, including genetic mutations, immune dysregulation, environmental exposures, and microbial infections [1, 2]. One hypothesis regarding the pathogenesis of CTCL suggests that it arises from antigenic stimulation in genetically susceptible individuals [2, 3], with evidence indicating that MF may develop in the context of chronic inflammation [2–4]. Specific HLA alleles have been implicated in an increased risk of developing MF and may affect prognosis, such as HLA-DQB1*∗*05, which is associated with the poorest prognosis [5].

In recent years, the role of the human microbiota has gained increasing attention as a contributing factor to CTCL. The microbiota, comprised of microorganisms inhabiting various body sites including the skin, gut, oral cavity, and genital tract, interacts with the host immune system, influencing its development and function. Dysbiosis, an imbalance in the composition or function of the microbiota, has been associated with a range of inflammatory and neoplastic diseases, further highlighting the potential connection between the microbiota and CTCL [5]. Several studies have suggested that dysbiosis may play a role in the pathogenesis or prognosis of CTCL. Some of these studies have reported alterations in the skin microbiota of patients with MF or SS when compared to healthy controls or patients with other dermatoses [6–8]. Other studies have found association between gut microbiota dysbiosis and systemic inflammation or immune suppression in patients with CTCL [9]. Moreover, some studies have explored the potential therapeutic effects of modulating the microbiota in CTCL, such as using antibiotics [10–12]. However, the evidence on this topic is still scarce, inconsistent, and limited by methodological issues. The aim of this systematic review is to summarize the current literature on the relationship between human microbiota and CTCL, focusing on its etiopathogenic implications and possible new therapeutic targets.

## 2. Methods

This systematic review was conducted following the Preferred Reporting Items for Systematic Reviews and Meta-Analyses (PRISMA) guidelines [13].

### 2.1. Search Strategy

**Figure 1:**
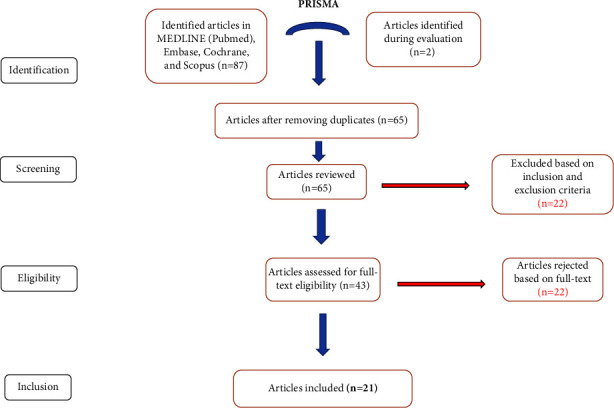
PRISMA flow diagram of the study selection process for the systematic review and meta-analysis of cutaneous T-cell lymphoma and microbiota. The diagram shows the number of records identified, screened, included, and excluded at each stage of the review, as well as the reasons for exclusion. Adapted from PRISMA flow diagram.

From January 2013 to March 2023, we searched the following electronic databases: MEDLINE (PubMed), Embase, The Cochrane Library, and Scopus. We used a combination of controlled vocabulary terms and free-text words related to “lymphoma, T-cell, cutaneous,” “microbiota,” “Mycosis Fungoides,” “Sézary Syndrome,” “lymphoma, primary cutaneous anaplastic large cell,” “Lymphomatoid Papulosis” and “Microbiota,” “microbiome,” “Microbial Community,” and “Microbial Communities.” We also applied a filter for human studies. The detailed search strategy for each database is provided in Figure 1. We also screened the reference lists of the included studies and relevant reviews for additional articles.

### 2.2. Study Selection

One reviewer screened the titles and abstracts of the retrieved records for eligibility. Discrepancies were resolved by discussion or by consulting a second and third reviewer. The full texts of potentially eligible articles were obtained and assessed for inclusion by the same three reviewers. The reasons for the exclusion of full text articles were recorded.

#### 2.2.1. Inclusion Criteria

We included original research articles that met the following criteria: (i) reported data on human microbiota (skin, gut, or other body sites) in patients with CTCL (any subtype) or healthy controls; (ii) used molecular methods (such as 16S rRNA gene sequencing or metagenomics) to characterize the microbiota; (iii) provided quantitative or qualitative results on microbiota composition, diversity, or function; and (iv) published in English or Spanish.

#### 2.2.2. Exclusion Criteria

We excluded articles that met any of the following criteria: (i) not original research articles (such as reviews, editorials, or case reports); (ii)used only culture-based methods to characterize the microbiota; (iii) did not provide sufficient data on microbiota analysis; and (iv) were duplicates or overlapping publications.

## 3. Results

### 3.1. Study Selection

The flow diagram of the study selection process appears in Figure 1. A total of 87 records were identified from the database searches. After removing 12 duplicates, 75 records were screened by title and abstract. Of these, 31 records were excluded for not meeting the inclusion criteria. The full texts of the remaining 44 records were assessed for eligibility. Among these, 23 records were further excluded for various reasons (see exclusion criteria). Finally, 21 studies (see Table 1) were included in this systematic review.

### 3.2. Study Characteristics

The main characteristics of the included studies are summarized in Table 1. These studies were published between 2013 and 2022, with most of them (*n* = 14) published after 2019. Most articles have focused on *Staphylococcus aureus* and its pathogenic explanation in patients with cutaneous T-cell lymphoma, with an emphasis on classic mycosis fungoides and Sézary syndrome. Other studies explored the potential application of antibiotic therapy or antiseptics as targeted treatments for cutaneous T-cell lymphoma. Additional articles investigated the cutaneous microbiome and the differences between lesional and nonlesional skin. Finally, one article examined the differences in gut microbiota between patients with T-cell lymphoma and healthy individuals.

### 3.3. Cutaneous Microbiota Composition

Microbiota composition (Table 2) refers to the relative abundance of different microbial taxa at various levels of resolution such as phylum, class, order, family, genus, or species. Most studies reported differences in microbiota composition between patients with CTCL and healthy controls or other comparators [14–17]. However, the results were inconsistent across studies, and no clear patterns emerged [18].

Salava et al. [3] investigated the skin microbiome of 20 patients diagnosed with stage IA-IIB mycosis fungoides using 16S rRNA and whole-genome sequencing on skin swab samples. Control samples were collected from healthy-looking skin on the opposite side of the same individual. The study did not find any significant differences in microbial diversity or at the genus level. Though in WGS data analysis, the authors noted a higher abundance of *Staphylococcus argenteus* in lesional skin, further investigation showed this observation to be inaccurate. Nonetheless, the study did reveal ten bacterial species (*Streptomyces* sp. *SM17*, *Bordetella pertussis*, *Streptomyces* sp. *PVA 94-07*, *Methylobacterium oryzae*, *Serratia* sp. *LS-1*, *Burkholderia mallei*, *Enterobacteriaceae bacterium*, *Achromobacter ruhlandii*, *Pseudomonas* sp. *A214*, and *Pseudomonas* sp. *st29*) that was more abundant in nonlesional skin.

Harkins et al. [4] collected samples from the nares, lower back, and thigh skin (sites of CTCL predilection) using premoistened swabs, as well as air controls. These samples were analyzed through shotgun metagenomic sequencing. The study found no significant differences in the abundance of viruses and fungi between lesional samples and healthy volunteers, or between Mycosis Fungoides and Sézary Syndrome patients. However, they found higher relative abundances of *Corynebacterium* spp. and lower relative abundances of *Cutibacterium* spp. in CTCL patients.

Dehner et al. [2] utilized 16S rRNA gene sequencing to analyze skin swab samples and observed the presence of *Bacillus safensis*, a rare human skin commensal found only in individuals with diagnosed CTCL, in skin swabs collected from lesional skin of the extremities of CTCL patients but not in other samples. They also isolated T-cells and tested the cytokine concentration and proliferative response to different bacteria, finding that only *B. safensis* stimulated the proliferation of T-cells.

Zhang et al. [1] utilized 16S rRNA gene sequencing to analyze skin swab samples and found no significant differences in bacterial diversity and richness between lesional and nonlesional skin. However, they observed higher abundance of *Corynebacterium* spp. and *Neisseriaceae* in lesional skin, whereas nonlesional samples were characterized by an increased abundance of *Sandaracinobacter* spp. and *Enhydrobacter* spp. They also found microbiome alterations depending on the disease phenotype, with an increase in *Staphylococcus* spp. observed in patients with marked erythema and a decrease in *Propionibacterium* spp. and *Bradyrhizobium* spp. in thickened skin. Painful lesions were associated with decreased *Propionibacterium* spp., increased *Bradyrhizobium* spp., and *Staphylococcus* spp. Excoriation was characterized by reduced *Conchiformibus* spp., and pruritus was associated with an increase in *Sphingomonas* spp. and *Parvimonas* spp.

Hooper et al. [6] studied the effect of ultraviolet light on the skin microbiome in lesioned (LS) and nonlesioned (NLS) skin of patients with CTCL, revealing that UV exposure alters the abundance of certain bacteria in both lesioned and nonlesioned skin. In summary, the data shows that UV exposure returns the microbial communities of lesioned skin to a more normal-like state in responsive patients, while in nonresponsive patients; the skin displays signs of ongoing dysbiosis.

### 3.4. *Staphylococcus aureus*

*Staphylococcus aureus* has been associated with mycosis fungoides and its variants, as its presence and enterotoxin generate a microenvironment that could influence the progression of CTCL [6, 11, 20–24]. The enterotoxin acts on both healthy lymphocytes and lymphomatous cells, stimulating the secretion of cytokines (IL-2, IL-17, and IL-21) that activate the JAK-STAT3 and JAK-STAT5 pathways, which are hyperactive in CTCL [21, 24–26]. Unlike normal conditions, in CTCL, the activation of these pathways is constant and elevated.

**Figure 2:**
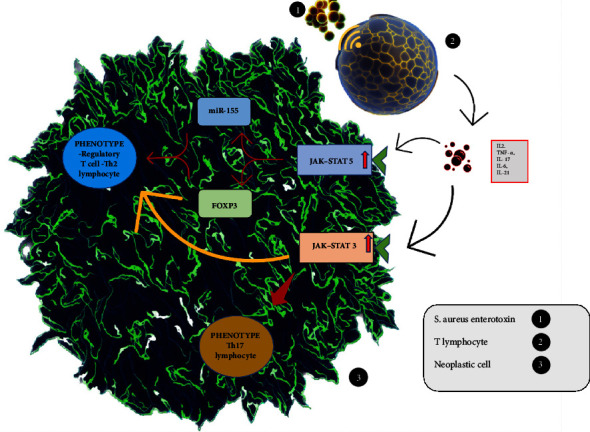
Effects of *Staphylococcus aureus* and its enterotoxin on the immune response in cutaneous T-cell lymphoma. The figure illustrates how the bacterial infection and its toxin activate JAK-STAT3 and JAK-STAT5 pathways, leading to the generation of regulatory T cells (Treg), TH2 cells, and Th17 cells, which suppress the antitumor immunity and promote chronic inflammation and genomic instability. Adapted from [23–26].

*Staphylococcus aureus* and its enterotoxin (Figure 2) induce activation of JAK-STAT3 and JAK-STAT5 induces a regulatory T-cell (Treg) phenotype through increased expression of the pro-oncogene FOXP3 [23]. This phenotype releases IL-10 and TGF-*β*, promoting immunotolerance. Furthermore, the hyperactivation of miR-155 by STAT5 contributes to a TH2 phenotype [24], which, through its cytokines (IL-4, IL-5, and IL-13), inhibits the TH1 response [22, 24], preventing an adequate defense of the organism against the tumor. On the other hand, STAT3 promotes a Th17 response with the release of IL-17 and IL-22, perpetuating chronic inflammation [25, 26]. Also, chronic inflammation promotes an increase in chromosomal aberrations that enhance the activity of STAT3 and STAT5, or alterations leading to the progression of the lymphoma, such as TP3, MYC, and PTEN [20, 23].

Lindahl et al. [16] observed an improvement in skin condition after treatment with intravenous antibiotics (cephalosporins and metronidazole) in patients with stage IIB CTCL. Interestingly, they also noted a decrease in cell proliferation and expression of interleukin-2 receptor (IL2R)-a and tyrosine-phosphorylated STAT3 (pYSTAT3) in immunohistochemical staining after treatment [12]. However, long-term use of antibiotics in CTCL patients can promote the development of resistant strains of *S. aureus*, leading to greater challenges in future eradication efforts. Therefore, long-term antibiotic therapy should only be considered in selected patients and not used daily [12]. Another proposed treatment regimen, the Duvic regimen, consisting of intravenous vancomycin and cefepime combined with antiseptic whirlpool baths and corticosteroids with alternating topical antibiotics, resulted in significant improvements in the skin condition of patients with erythrodermic CTCL and *S. aureus* colonization [7, 10]. Genetic methods for bacterial identification remain expensive, and standard bacterial cultures could be a useful tool for identifying individuals who require antibiotics. Patients with CTCL could also benefit from nonantibiotic therapies to eradicate bacterial colonization. Bleach baths have been found to be a helpful treatment option for patients with atopic dermatitis, with lower *S. aureus* abundance observed and improvements in skin condition following *S. aureus* eradication [10, 12]. Further studies are needed to determine the effectiveness of bleach baths on the skin microbiome in other disease entities.

### 3.5. Gut Microbioma

Only one study focusing on gut microbiota was included in our systematic review. Hooper et al. [9] investigated the gut microbial profiles of 38 patients with cutaneous T-cell lymphoma (CTCL) and 13 healthy individuals using 16S rRNA gene amplicon sequencing. The results indicated that bacterial dysbiosis was more pronounced in CTCL patients with advanced disease. The study is significant because it is the first to characterize the gut microbiome in CTCL and is one of the largest sample sets for this disease. Notably, the study found that certain bacterial populations, including *Coriobacteriaceae*, *Lactobacillus*, and *Bifidobacterium*, which were reduced in CTCL patients, have been shown to have beneficial effects in the gut. Bacteria from the family *Coriobacteriaceae* are known to strengthen gut barrier function, species from the genus *Lactobacillus* are capable of cytokine-based anti-inflammatory activity, and species from the genus *Bifidobacterium* may promote daily colonic epithelial renewal. In addition, the study found that the presence of *Ruminococcaceae* was inversely correlated with IL-6 and C-reactive protein levels.

The study also noted that the dysbiotic signatures observed in CTCL, as well as other malignancies such as psoriasis, may be explained by the complex interactions between the gut microbiome and the immune system. Certain bacterial populations, such as *Prevotellaceae* and *Bacteroidaceae*, have been shown to promote local and distant Th17 differentiation and subsequent IL-17 release, which may contribute to CTCL pathogenesis. The study also found that the loss of butyrate-producing species and enrichment of lipopolysaccharide-secreting species have been linked to tumor proliferation, and that CTCL samples were associated with the loss of butyrate producers and Proteobacteria dysbiosis.

## 4. Discussion

This systematic review aimed to summarize the current evidence on the human microbiota in patients with cutaneous T-cell lymphoma (CTCL) compared to healthy controls or other comparators. We included 21 studies [6–12, 14–17, 19–28] that analyzed the microbiota from different body sites using molecular methods. The principal finding is that there is no clear consensus on the microbiota's association with CTCL, with most studies focusing almost exclusively on mycosis fungoides (MF) and Sézary syndrome (SS). The observed discrepancies can be attributed to the heterogeneity of study designs, including variations in sample size, sampling sites, sequencing methods, bioinformatics pipelines, and statistical analyses. Moreover, most of the studies were cross-sectional and observational, which limits the causal inference and generalizability of the results. Therefore, more standardized and rigorous studies are needed to establish a reliable and reproducible microbiota profile for patients with CTCL.

Cutaneous microbiota composition, diversity, and function were differentially altered in patients with CTCL type MF and SS, which could play a role in their development, but the results were inconsistent and conflicting across studies [14–18]. There is substantial evidence supporting the association between *Staphylococcus aureus* and on MF and SS. The effect of antibiotics on the colonization of *S. aureus* in patients with on MF and SS has been studied, with positive clinical outcomes observed after antibiotic treatment [29–31]. Talpur et al. [31] identified *S. aureus* colonization in patients with MF and SS, and subsequent treatment with dicloxacillin, nafcillin, ampicillin, cephalexin, or clindamycin resulted in negative skin cultures in 30 out of 33 patients after four to eight weeks. There is some evidence on the usefulness of antibiotics and antiseptics for MF and SS clinical improvements, as well as improvements in various severity markers of MF and SS [10–12, 27].

Gut dysbiosis has been linked to cancer and inflammatory skin diseases. Research has revealed the tumorigenic potential of certain bacterial taxa and their potent immunomodulatory capabilities [32]. This suggests that intestinal dysbiosis might both encourage and reflect more severe immune dysfunction. It is also understood that intestinal dysbiosis contributes to systemic disease through cytokine-induced inflammation, abnormal activation of effector T-cells, and disruptions in the intestinal epithelial barrier that lead to bacterial translocation from the intestinal lumen [33–35]. Although Hooper et al. [9] provide preliminary evidence suggesting that the gut microbiota may be implicated in on MF and SS; we cannot draw definitive conclusions at this time due to the limitations of this single, preliminary study. Some of its limitations include a small patient population, heterogeneity among patients, and the fact that, although patients who had received antibiotic therapy within the last month were excluded, we cannot rule out the possibility of permanent changes in the gut microbiome resulting from antibiotic therapy prior to that exclusion period. Therefore, it is necessary to conduct further studies with larger, more homogeneous patient populations and comparison groups, considering any antibiotic therapy received.

The interaction between tumor cells and the microenvironment influences the progression of MF and SS. In early-stage MF, neoplastic cells are scarce, and reactive helper T-lymphocytes (Th1) and CD8+ T-lymphocytes contribute to antitumor defense [2–4]. As disease progresses, the tumor microenvironment shifts from a Th1 to a Th2 response, contributing to tumor cell growth and immune escape [2, 36].

Mechanistic studies are also needed to explore how the microbiota may influence the pathogenesis, progression, or treatment response of MF and SS. Some possible mechanisms that have been proposed are: (i) The microbiota may modulate the immune system and inflammation [32–35]; (ii) the microbiota may produce metabolites or toxins that affect the skin barrier function [33–35, 37]; (iii) the disruption of the skin barrier promotes cutaneous dysbiosis [6, 26]; (iv) cutaneous dysbiosis leads to chronic skin inflammation [6, 14–17, 26]; (v) chronic inflammation leads to continuous overstimulation, which results in the accumulation of genetic abnormalities. These abnormalities, in turn, cause overstimulation of STAT5 and STAT3 (Figure 2), creating a self-perpetuating cycle [6–8, 20–22, 25, 28]; and (vi) chronic skin inflammation can result in MF or SS [21, 23–26, 38]. However, these hypotheses need to be experimentally tested in animal models or clinical trials. Furthermore, the potential role of other microbial kingdoms such as fungi, viruses, or archaea in CTCL remains largely unexplored.

This review pinpoints towards the role of the microbiota in CTCL and towards new insights into the etiology, diagnosis, prognosis, and therapy of CTCL. Some of these insights are: there is potential to use JAK-STAT inhibitors to block their overactivity in cutaneous T-cell lymphoma [39]. IL-17 has been implicated in the early and intermediate stages of this lymphoma, where it contributes to chronic inflammation and epidermotropism, making it a possible target for therapy [26]; the microbiota may serve as a biomarker for early detection or risk stratification of MF and SS [7, 9, 10, 12, 27]; the microbiota may be a target for novel therapeutic interventions such as probiotics, prebiotics, symbiotic, or fecal transplantation [9]; the microbiota may be a modifier for existing treatments such as chemotherapy, immunotherapy, or phototherapy [7, 9, 10, 12, 19, 27]. However, these possibilities need to be validated by further research before they can be translated into clinical practice.

## 5. Conclusion

Although studies on the composition of the cutaneous and gut microbiota in CTCL patients have shown inconsistent and varied results, it seems that microbial dysbiosis plays a role in the disease, particularly in cases of mycosis fungoides and Sézary syndrome, with insufficient research conducted on other types of cutaneous T-cells. The relationship between *Staphylococcus aureus* and mycosis fungoides progression is a promising area of research, and antibiotic treatments might offer clinical improvements in the skin of affected patients. However, more studies are needed to fully understand the interaction between the cutaneous and gut microbiota and mycosis fungoides, as well as to investigate alternative treatments that can eradicate bacterial colonization without contributing to antibiotic resistance and without eliminating commensal bacteria with positive impact on patient's health.

Looking ahead, it would be intriguing to explore the utility of anti-JAK and anti-TNF-17 inhibitors in certain phases of the disease, potentially opening new avenues for treatment.

## Figures and Tables

**Table 1:** Articles included in the systematic review of cutaneous T-cell lymphoma and microbiota.

#	Authors	Title	Main findings
17	Zhang et al.	TCL-036: Alternations in the Skin Microbiota Are Associated with Symptom Severity in Mycosis Fungoides	Skin microbiota alterations are linked to symptom severity in mycosis fungoides
16	Dehner et al.	Skin Microbiota as Antigenic Triggers for Cutaneous T Cell Lymphoma	Skin microbiota may act as antigenic triggers for CTCL
14	Salava et al.	Skin Microbiome in Cutaneous T-Cell Lymphoma by 16S and Whole-Genome Shotgun Sequencing	Investigated skin microbiome in CTCL using 16S and whole-genome shotgun sequencing
15	Harkins et al.	Cutaneous T-Cell Lymphoma Skin Microbiome Characterized by Shifts in Certain Commensal Bacteria	CTCL skin microbiome shows shifts in certain commensal bacteria compared to healthy controls
10	Lewis	Cutaneous microbiota in the pathogenesis of cutaneous T-cell lymphoma and role of antibiotic therapy	Explores the role of cutaneous microbiota and antibiotic therapy in CTCL pathogenesis
19	Hooper et al.	Skin microbiome in cutaneous T-cell lymphoma associated with phototherapy treatment response	Skin microbiome in CTCL is linked to phototherapy treatment response
25	Wu & Hwang	A Microbiota-Dependent, STAT3-Driven Mouse Model of Cutaneous T-Cell Lymphoma	Describes a microbiota-dependent, STAT3-driven mouse model of CTCL
37	Fanok et al.	Analysis of molecular etiology and bacterial triggers of cutaneous T-cell lymphoma	Investigates molecular etiology and bacterial triggers of CTCL
6	Kadin et al.	Evidence linking atopy and staphylococcal superantigens to lymphomatoid papulosis pathogenesis	Links atopy and staphylococcal superantigens to lymphomatoid papulosis pathogenesis
9	Hooper et al.	Gut dysbiosis in CTCL characterized by shifts in bacterial taxa and decreased diversity	Gut dysbiosis in CTCL shows shifts in bacterial taxa and decreased diversity in advanced disease
7	Emge et al.	MRSA is an important pathogen in erythrodermic CTCL patients	MRSA plays a significant role in erythrodermic CTCL patients
8	Fujii	Pathogenesis of cutaneous T-cell lymphoma: Involvement of *Staphylococcus aureus*	*Staphylococcus aureus* involvement in CTCL pathogenesis
20	Fanok et al.	Role of Dysregulated Cytokine Signaling and Bacterial Triggers in CTCL Pathogenesis	Dysregulated cytokine signaling and bacterial triggers play a role in CTCL pathogenesis
21	Krejsgaard et al.	Staphylococcal enterotoxins stimulate lymphoma-associated immune dysregulation	Staphylococcal enterotoxins contribute to lymphoma-associated immune dysregulation
22	Blümel et al.	*Staphylococcus aureus*alpha-toxin inhibits CD8 (+) T-cell-mediated killing of cancer cells in CTCL	*S. aureus*alpha-toxin hinders CD8 (+) T-cell-mediated killing of cancer cells in CTCL
27	Lindahl et al.	*Staphylococcus aureus* and Antibiotics in Cutaneous T-Cell Lymphoma	*S. aureus* and antibiotics' role in CTCL
23	Willerslev-Olsen et al.	*Staphylococcus aureus* enterotoxins induce FOXP3 in neoplastic T-cells in Sézary syndrome	*S. aureus* enterotoxins induce FOXP3 expression in neoplastic T-cells in Sézary syndrome
24	Willerslev-Olsen et al.	*Staphylococcus aureus* Induces STAT5-Dependent miR-155 Expression in Cutaneous T-Cell Lymphoma	*S. aureus* induces STAT5-dependent miR-155 expression in CTCL
11	Lewis et al.	The “Duvic Regimen” for Erythrodermic Flares Secondary to *Staphylococcus aureus* in Mycosis Fungoides	Describes the “Duvic Regimen” for treating erythrodermic flares caused by *S. aureus* in mycosis fungoides
26	Willerslev-Olsen et al.	Staphylococcal enterotoxin A (SEA) stimulates STAT3 activation and IL-17 expression in CTCL	SEA activates STAT3 and induces IL-17 expression in CTCL
12	Lindahl et al.	Antibiotics inhibit tumor activity and disease in cutaneous T-cell lymphoma	Antibiotics inhibit tumor activity and disease progression in CTCL

**Table 2:** Summary of articles describing the cutaneous microbiota in patients with cutaneous T-cell lymphoma (CTCL). The table shows the main findings and methods of each study.

Study	Type of sample	Method of analysis	Summary	Key finding
17. Zhang et al. (2021)	Skin swabs from mycosis fungoides patients	16S rRNA gene sequencing	Analysis of bacterial diversity in lesional and nonlesional skin and relation to CTCL symptoms	Alterations in the skin microbiota are associated with symptom and phenotype in mycosis fungoides
16. Dehner et al. (2018)	Skin samples from cutaneous T-cell lymphoma patients	16S rRNA gene sequencing	Investigates the role of skin microbiota as antigenic triggers for cutaneous T-cell lymphoma	Presence of *Bacillus safensis* only in CTCL patients
14. Salava et al. (2020)	Skin swabs from cutaneous T-cell lymphoma patients	16S rRNA and whole-genome sequencing	Explores the skin microbiome in cutaneous T-cell lymphoma using 16S rRNA and whole-genome shotgun sequencing	Cutaneous T-cell lymphoma patients have distinct skin microbiome profiles compared to healthy controls. 10 bacterial species more abundant in nonlesional skin^*∗*^
15. Harkins et al. (2021)	Skin samples from cutaneous T-cell lymphoma patients	Shotgun metagenomic sequency	Examines the cutaneous T-cell lymphoma skin microbiome and its shifts in certain commensal bacteria.	Higher abundance of *Corynebacterium* spp. and lower abundance of *Cutibacterium* spp. In CTCL patients
19. Hooper et al. (2022)	Cutaneous microbial changes in CTCL patients treated with UV	16S gene sequencing	Examination of microbial changes in the skin of CTCL patients treated with UV, noting differences in microbial richness and in microbial communities in response to	Differences in UV treatment response between responders and nonresponders, with specific changes in the abundance of certain bacteria.

^*∗*^ *Streptomyces* sp. SM17, *Bordetella pertussis*, *Streptomyces* sp. PVA 94-07, *Methylobacterium oryzae*, *Serratia* sp. LS-1, *Burkholderia mallei*, *Enterobacteriaceae bacterium*, *Achromobacter ruhlandii*, *Pseudomonas* sp. A214, and *Pseudomonas* sp. st29.

## Data Availability

The data supporting the conclusions of this study were compiled from the systematic review detailed in the article. The sources of this data are articles retrieved from the following bibliographic databases: MEDLINE (PubMed), Embase, The Cochrane Library, and Scopus. All referenced articles were published within the last 10 years. As the data were derived from these published sources, they are accessible through the respective databases. Any data that are not available is due to restrictions or limitations imposed by the original publication source.
